# Be prepared for intraoperative anaphylaxis

**DOI:** 10.4103/0019-5049.63630

**Published:** 2010

**Authors:** SK Dube, GR Agrawal, BN Pratihary, R Dutta, RK Verma

**Affiliations:** Department of Anaesthesiology, Institute of Medical Sciences, Banaras Hindu University, Varanasi-221 005, India

Sir,

A 30-yr-old female presented with a gradually progressive swelling on the right side of the neck for 7 yrs along with tingling, numbness and pain in the right upper limb for 1 yr. She had a 6 cm × 5 cm smooth, firm, globular, nontender mass in the right supraclavicular region without any local rise of temperature, venous engorgement or skin change. Her airway examination was normal. On examination she was found to have sensory loss in the medial side of the arm, forearm and the medial half of the palm and dullness and diminished breath sound in the right apical lung area.

Routine blood investigations, Electrocardiogram (ECG) and ultrasound of the abdomen were within normal limits. Chest X-ray revealed an ill-defined mass in the right supraclavicular region. Magnetic resonance imaging (MRI) of the thoracic region showed a well-defined 8.26 cm × 6.36 cm × 4.30 cm mass in right para-vertebral region extending from C7 to T2 vertebral levels along with intraspinal extension and cord compression. Cervical intervertebral disc and airway anatomy were normal. A provisional diagnosis of neurogenic tumor was made and the case was posted for excision under general anaesthesia (GA).

After connecting the pulse oximeter, ECG electrodes and blood pressure cuff, we established a 14G intravenous line and premedicated the patient with intravenous (IV) fentanyl 2 *μ*g/kg and ondansetron 0.1 mg/kg. GA was induced with IV propofol 2 mg/kg and succinyl choline 1.5 mg/kg IV and the trachea was intubated with an appropriate sized flexometallic tube. We cannulated the left internal jugular and radial artery after induction of GA. Anaesthesia was maintained with isoflurane and injection of vecuronium and fentanyl.

**Figure 1:**
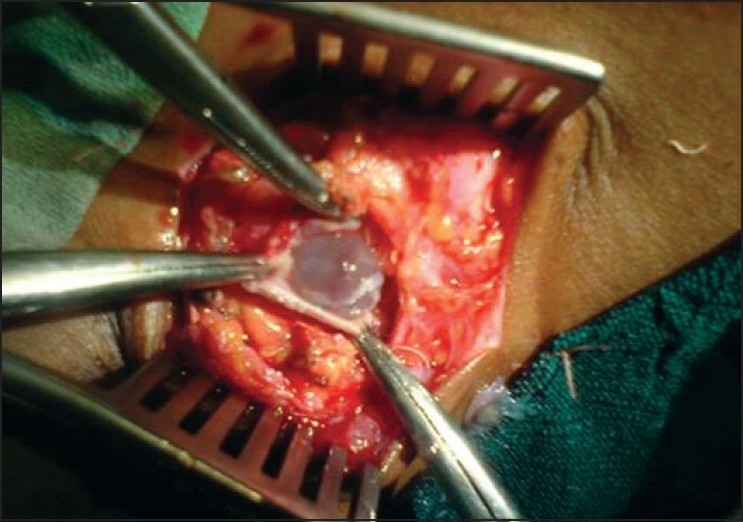
Photograph of operative field showing hydatid cyst

After 45 min of stable vitals of the patient, we noticed a sudden drop of blood pressure, tachycardia and increase in airway pressures. We suspected an anaphylactic reaction which was further supported by reporting of an accidentally punctured hydatid cyst by the surgeon [Figure 1]. Immediately, all the anaesthetic gases were stopped and the patient was ventilated with 100% oxygen followed by administration of IV epinephrine 0.2 *μ*g/kg, hydrocortisone 5 mg/kg and 2 l of Ringer's lactate solution. In view of the persistent hypotension, we put the patient on epinephrine infusion at a rate of 0.005 μg /kg/min for a short duration. After 20 min, the patient's vitals and airway pressure returned to normal. Rest of the surgery was uneventful.

Hydatid disease or echinococcosis is caused by infestation of one of the three species of the genus *Echinococcus*. The location of hydatid cyst is mostly hepatic (75%) and pulmonary (15%) and only 10% occurs in the rest of the body, i.e., brain, heart, kidney, ureter, spleen, uterus, fallopian tube, mesentery, pancreas, diaphragm and muscles.[1,2] However, hydatid cyst rarely occurs in the supraclavicular area. The allergic reactions caused by the strongly antigenic hydatid fluid released due to episodic leakage or rupture of the hydatid cyst in sensitized individuals, may range from fever, pruritus, urticaria, to frank anaphylactic shock.[3]

Cardiovascular symptoms (73.6%), cutaneous symptoms (69.6%) and bronchospasm (44.2%) are the most common manifestations of anaphylaxis during anaesthesia.[4] Intraoperative anaphylaxis often manifests as bronchospasm and cardiovascular collapse because early cutaneous signs of anaphylaxis are masked by sedation, unconsciousness and poor exposure. Intraoperative anaphylaxis has a high mortality rate if not diagnosed and treated early. Moreover, anaphylaxis is often an unanticipated phenomenon and prevention is the most important component to decrease the incidence of anaphylaxis.[5] Hence, an early anticipation can lead to a prompt diagnosis and management of anaphylaxis. In regions where hydatid disease is endemic, the possibility of hydatid cyst at an uncommon site and the associated risk of anaphylaxis should always be kept in mind whenever dealing with such cases.
